# Floating Hydrogel with Self-Generating Micro-Bubbles for Intravesical Instillation

**DOI:** 10.3390/ma9121005

**Published:** 2016-12-12

**Authors:** Tingsheng Lin, Xiaozhi Zhao, Yifan Zhang, Huibo Lian, Junlong Zhuang, Qing Zhang, Wei Chen, Wei Wang, Guangxiang Liu, Suhan Guo, Jinhui Wu, Yiqiao Hu, Hongqian Guo

**Affiliations:** 1 Department of Urology, Drum Tower Hospital, Medical School of Nanjing University, Institute of Urology, Nanjing University, Nanjing 210008, China; m18260087829@163.com (T.L.); dr.zxz@hotmail.com (X.Z.); nandalhb@sina.com (H.L.); zhuangjl-2008@163.com (J.Z.); drzhangq@163.com (Q.Z.); chenwei1213@gmail.com (W.C.); wawe9999@163.com (W.W.); liuguangxiangnju@163.com (G.L.); dr.ghq@163.com (S.G.); 2 State Key Laboratory of Pharmaceutical Biotechnology, Nanjing University, Nanjing 210093, China; cyanii@yahoo.cn

**Keywords:** floating hydrogel, shaking, thermo-sensitive hydrogel, intravesical instillation, acute bladder injury

## Abstract

Intravesical instillation is the main therapy for bladder cancer and interstitial cystitis. However, most drug solutions are eliminated from bladder after the first voiding of urine. To solve this problem, we proposed a floating hydrogel with self-generating micro-bubbles as a new delivery system. It floated in urine, avoiding the urinary obstruction and bladder irritation that ordinary hydrogels caused. In this study, we abandoned traditional gas-producing method like chemical decomposition of NaHCO_3_, and used the foamability of Poloxamer 407 (P407) instead. Through simple shaking (just like shaking SonoVue for contrast-enhanced ultrasound in clinical), the P407 solution will “lock” many micro-bubbles and float in urine as quickly and steadily as other gas producing materials. In vivo release experiments showed that drug was released continually from hydrogel for 10 h during the erosion process. Thus, the residence time of drug in bladder was prolonged and drug efficacy was improved. In vivo efficacy study using rabbit acute bladder injury model showed that prolonged drug residence time in bladder increased the efficiency of heparin in the protection of bladder mucosal permeability. Therefore, our floating hydrogel system with self-generating micro-bubbles was single-component, simply prepared and efficacy enhancing, successfully exempting users from worries on safety and clinical efficiency from bench to bedside.

## 1. Introduction

Diseases of the urinary bladder account for a large number of severe medical conditions affecting patients of all ages. The major diseases affecting the urinary bladder are bladder cancer and intractable interstitial cystitis. Intravesical instillation is the most common treatment for the bladder cancer and intractable interstitial cystitis [1]. It is performed by instilling chemical drugs to bladder using a urethral catheter. The efficacy of the intravesical treatment depends on the residence time of drug inside the bladder, which relates to the adhesive capability of the drug onto the urinary bladder wall [2]. However, most drug solutions were eliminated during the first voiding of urine after intravesical instillation. The efficacy of intravesical instillation relies on the drug residence time in bladder, so it is imperative to develop a controlled drug delivery system.

Recently, hydrogel had been used as drug reservoir to extend the drug residence time in bladder [3,4,5]. However, due to the high viscosity of hydrogel, one serious problem is urinary obstruction. Gels adhering to the bladder may block the urinary tract, such as internal urethral orifice and ureteric orifice with narrow opening. As hydrogels attach to the bladder wall, another problem is serious bladder irritation symptoms, such as blood clots in bladder [6]. The two floating hydrogel drug delivery systems we developed before were able to overcome these two shortcomings and prolonged the resident time of drug in the bladder [7,8]. The first floating hydrogel system consisted of thermo-sensitive polymer (Poloxamer 407) and NaHCO_3_, which was liquid at low temperature while forming gel at body temperature (37 °C). In the presence of H^+^, NaHCO_3_ decomposed and produced CO_2_, which attached to the surface of hydrogel and float the hydrogel in urine. Hence, the urinary tract will not be blocked, and the encapsulated drug released in a controlled manner. However, we need to acidify the urine to provide enough acid environment (pH < 5.6) for hydrogel floating in urine. To realize urine acidification, patients have to take oral drugs, like ammonium chloride and vitamin C. Extra drugs increase the risk of side effects including acid–base imbalance and electrolyte disturbances. Thus, the practical application of this floating hydrogel system was limited. In the second floating hydrogel system, NaHCO_3_ was substituted by Ammonium bicarbonate (NH_4_HCO_3_) as micro-bubbles producers. NH_4_HCO_3_ decomposed at body temperature without acidifying the urine and generated micro-bubbles inside hydrogel, which also float the hydrogel in urine. However, NH_4_HCO_3_ is unstable and easily decomposed, especially in the liquid solution, so this floating hydrogel system could not be stored for a long time. In addition, both NaHCO_3_ and NH_4_HCO_3_ are inorganic compounds, which might react with drugs in floating hydrogel and lead to chemical change of drugs. Therefore, more ideal floating scheme is required for the floating hydrogel system in practical clinical application.

**Figure 1:**
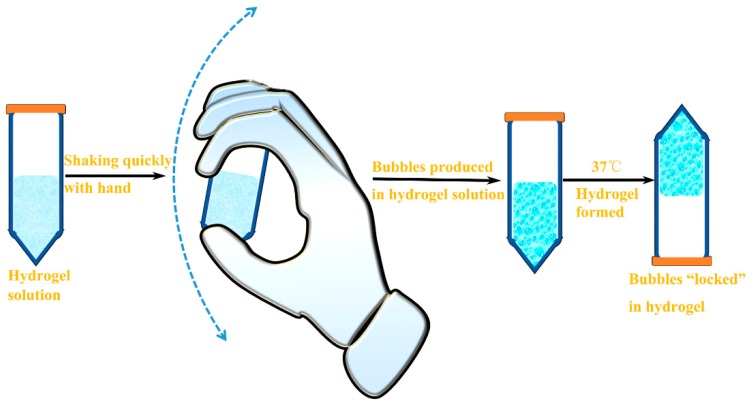
The schematic diagram of floating hydrogel preparation by shaking.

In this study, we developed a floating hydrogel drug delivery system merely with Poloxamer 407, with no additive for producing micro-bubbles. In fact, P407 solution could produce many micro-bubbles through shaking, stirring, or homogenizing, as P407 is a non-ionic surfactant [9]. In this system, no extra conditions, such as specific temperature and pH, were required; hence, safety and efficiency problems from bench to bedside were solved radically. By just shaking the hydrogel solution (like shaking SonoVue for contrast-enhanced ultrasound in clinical), micro-bubbles can be viewed as gas-filled micelles formed by surfactant molecules, whose hydrophobic tail groups face the hydrophobic gas and hydrophilic head groups face the aqueous phase [10,11]. Due to the viscosity of P407 solution, micro-bubbles were suspended for a certain amount of time and did not escape easily. Moreover, the micro-bubbles could be “locked” in hydrogel when the P407 solution converted to gel state at higher temperature due to its thermo-sensitive property (Figure 1). The P407 solution after shaking converted to gel and float in urine immediately after injected into bladder, and the encapsulated drug was released in a controlled manner. Our study showed that this floating hydrogel drug delivery system was easily prepared and safe. Because the production of micro-bubbles was milder and slower, the residence time of drug was extended and efficacy of intravesical drug delivery was improved.

## 2. Materials and Methods

### 2.1. Materials

Poloxamer 407 (P407) was purchased from Meilun Biology Technology Company, Ltd. (Dalian, China). Rhodamine B (RhB), Protamine sulfate (PS) and heparin were purchased from Sigma-Aldrich (St. Louis, MO, USA). Thirty New Zealand white male rabbits, weighting on average 2000 g (range, 1800–2500 g), were obtained from the Experimental Animal Center, University of Yangzhou, China. All animal protocols were approved by Institutional Animal Care and Use Committee (IACUC) of Nanjing University.

### 2.2. Preparation and Optimization of Floating Hydrogel

The non-floating hydrogel is the mixture of P407 and distilled water, and the P407 concentration was 45% (w/v). To prove that micro-bubbles could be produced by P407 solution and reserved in P407 solution, the P407 solutions was shaken by hand up and down at a speed of ≥2 round/s (Figure 1), and stored at 0 °C and 37 °C, respectively.

To determine the volume change of hydrogel in respect to the density of micro-bubble, 10 mL of P407 solution was shaken by hand for 0, 10, 20, 30, 40 times respectively. The volume changes of hydrogels after different shaking times were recorded. To determine the effect of environmental factors and shaking force on micro-bubble generation, the volume change of hydrogel in respect to the density of micro-bubble was measured. Ten milliliters of P407 solution was shaken by hand for 20 times at different environmental factors and the volume changes of hydrogels after shaking were recorded. To observe environmental effect, gel solutions were shaking at the local different temperature (3.5 °C, 11.5 °C, 25.3 °C and 31.9 °C), local different humidity (22.6%, 46.8%, 69.4% and 88.2%), local different atmosphere pressure (1005/100 Pa, 1009/100 Pa, 1013/100 Pa and 1015/100 Pa). To evaluate shaking force on micro-bubble generation, shaking speed in respect to the shaking force was measured. Shaking speed defined as the time of hand move up and down in one cycle (hand move up and down was one cycle, the distance of hand up or down was 50 cm).

To evaluate the effect of shaking time on micro-bubbles amount in hydrogel, P407 solution was shaken by hand for 0, 10, 20, 30, 40 times respectively. Micro-bubbles produced in hydrogel solution after different shaking time were recorded with photos and detected by ultrasound (B/K, Copenhagen, Denmark). To count the number of micro-bubbles, hydrogel was put on a glass slide and observed via a Zeiss M2Bio microscope (Zeiss, Oberkochen, Germany). The zoom of microscope was 40×, and images were captured. P407 solution after shaking was put in 37 °C water to observe its floating state.

### 2.3. Characterizations of Floating Hydrogel

To obtain information on the pore structure of hydrogels, the floating hydrogel shaken for 20 times was observed using Scanning electron microscopy. The samples were plunged in liquid nitrogen and freeze-dried to maintain the porous structure without any collapse. The samples were mounted on the base plate and coated with gold. The morphology was investigated using a Hitachi (Tokyo, Japan) S-570 Scanning Electron Microscope.

The bubble size distribution of the floating hydrogel was investigated using electron microscope (Nikon, Tokyo, Japan). The floating hydrogel after 20-times shaking was put on a glass slide, the zoom of microscope was 40×. Three fields were randomly selected to calculate the bubble size distribution.

The apparent viscosity was determined by a NDJ-1 viscometer (Shanghai Balance Instrument Factory, Shanghai, China). Twenty milliliters of hydrogel solution after 20-times shaking was put in a 25 mL beaker and placed in water bath. Then the solution was heated at a speed of 1 °C/min and the viscosity was recorded.

The erosion time was the time it takes for gel to dissolve completely in water. The volume of 5 mL, 10 mL, 15 mL, 20 mL floating hydrogels was injected into 37 °C water, and the erosion time of floating hydrogels were recorded.

The storage stability of the hydrogel of 45% P407 was also recorded. A digital timer (Thermo Fisher Scientific, Boston, MA, USA) was used for recording.

### 2.4. Incorporation of Drug in Floating Hydrogel

To study the release of floating hydrogel in vitro and in vivo, Rhodamine B was incorporated into hydrogel. A calculated amount of P407 (45%) was successively dissolved in RhB solution at 4 °C to form a RhB-loaded hydrogel solution. The concentration of RhB was 0.005% (w/v).

To study the efficacy of floating hydrogel in acute bladder injury model, Heparin was incorporated into hydrogel. A calculated amount of P407 (45%) was successively dissolved in Heparin solution at 4 °C to form a Heparin-loaded hydrogel solution. The concentration of Heparin was 1500 IU/mL.

### 2.5. Release Study In Vitro

The release study was performed in the dissolution tester (ZRS-8G, Instruments of Tianjin University, Tianjin, China). The PBS (pH = 7, 200 mL) in the beaker of dissolution tester was heated to 37 °C. RhB solution (50 ug/mL, 5 mL), RhB-loaded floating hydrogel (5 mL) and RhB-loaded non-floating hydrogel (5 mL) was injected into solution, respectively. At predetermined time points, 3 mL solution was collected and substituted with the same volume of fresh solution. The amount of RhB was determined by UV spectrophotometry (UV-2450, Shimadzu, Kyoto, Japan) at 555 nm.

### 2.6. Verification of Hydrogel Floating In Vivo

Rabbit was anesthetized with pentobarbital (30 mg/kg, intravenous injection). Floating hydrogel solution was intravesically instilled into bladder using a catheter. The floating hydrogel in bladder was detected by B ultrasound.

### 2.7. Release Study In Vivo

The rabbits were randomly chosen. They were maintained in a controlled atmosphere of 12 h dark/light cycle, 22 ± 2 °C temperature and 50%–70% humidity, with free access to pellet feed and fresh tap water. The animals were supplied with dry food pellets commercially available.

In fact, the gel solution was stored at 0 °C condition before injected to the bladder and the viscosity of 45% P407 was about 200 mPa·s at this temperature. The viscosity was low. Therefore, it is easy to inject the gel solution from the catheter to bladder. Rabbits were intravesically instilled with 3 mL normal saline, free RhB solution, RhB-loaded floating hydrogel and RhB-loaded non-floating hydrogel solution respectively. The RhB -loaded non-floating hydrogel solution did not undergo shaking. All animals were fastened on a desk. Intravesical administration was accomplished using a catheter (9F) inserted into the bladder through the urethra. Urine samples were collected. The concentration of RhB was determined by a fluorescence microplate reader (Safire, TECAN or Molecular Devices M3, Männedorf, Switzerland). The frozen section was immediately prepared after isolating rabbit bladder tissues. The fluorescence in the bladder section was observed using a Zeiss M2Bio fluorescence microscope. The thickness of frozen biopsy was 10 μm, and the exposure time of fluorescence microscope was 1 s.

### 2.8. The Efficacy of Floating Hydrogel in Acute Bladder Injury Model

Fifteen rabbits were randomly divided into five groups, with three rabbits for each group. All rabbits were fastened on the desk for intravesical instillation. For the control group, the bladders of rabbits were pretreated with 10 mL normal saline for 60 min, followed by 3 mL normal saline. For the PS + Saline group, the bladders of rabbits were pretreated with 10 mL Protamine sulfate (PS) (20 mg/mL) for 60 min, followed by 3 mL saline. For the PS + Gel group, the bladders of rabbits were pretreated with 10 mL PS (20 mg/mL) for 60 min, followed by 3 mL non-floating hydrogel (45% P407 hydrogel solution without shaking). For the PS + Heparin solution group, the bladders of rabbits were pretreated with 10 mL PS (20 mg/mL) for 60 min, followed by 3 mL heparin solution (1500 IU/mL). For the PS + Heparin loaded floating hydrogel group, the bladders of rabbits were pretreated with 10 mL PS (20 mg/mL) for 60 min, followed by 3 mL heparin loaded floating hydrogel (1500 IU/mL). After the treatments, all rabbits bladders were intravesically instilled 20 mL saline to trigger the first voiding of urine. After 10 h, all rabbits were intravesical instilled RhB solution (5 ug/mL) for 30 min. Then all rats were executed and bladder tissues were obtained. The frozen section was immediately prepared. The fluorescence in the bladder section was observed using a Zeiss M2Bio fluorescence microscope. The thickness of frozen biopsy was 5 μm, the exposure time of fluorescence microscope was 83 ms. The fluorescence depth of bladder tissues were calculated by the software Image-pro 6.0 (Media Cybernetics, Bethesda, MD, USA).

## 3. Results

### 3.1. Preparation and Optimization of Floating Hydrogel

**Figure 2:**
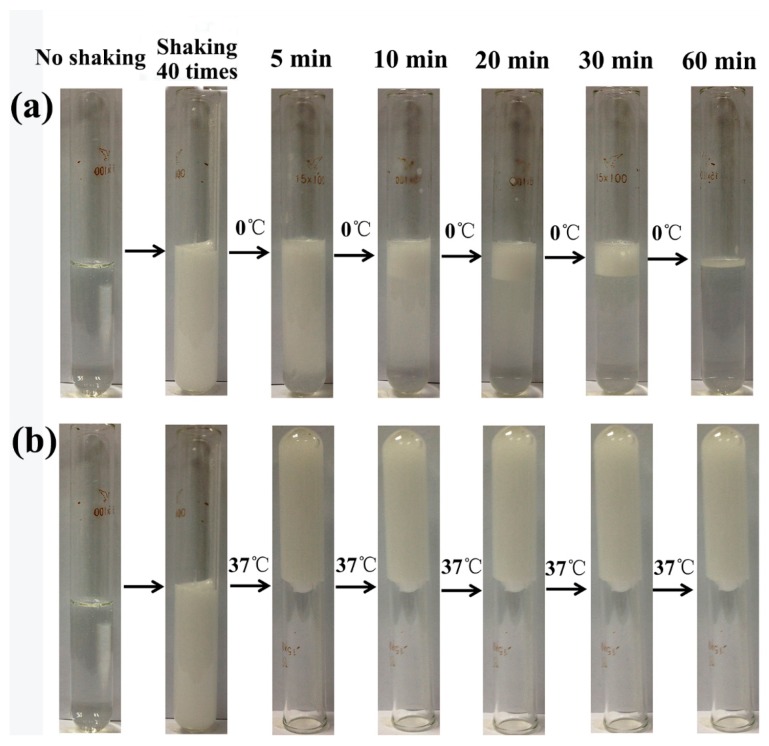
Bubbles in hydrogel solutions after shaking at 0 °C and 37 °C. To confirm that micro-bubbles were blocked in hydrogel at different temperature, hydrogel solutions after shaking were placed at 0 °C and 37 °C, respectively. The hydrogel could hold micro-bubbles in gel during the whole experiment at 37 °C (**a**). Micro-bubbles dissipated gradually at 0 °C (**b**).

**Figure 3:**
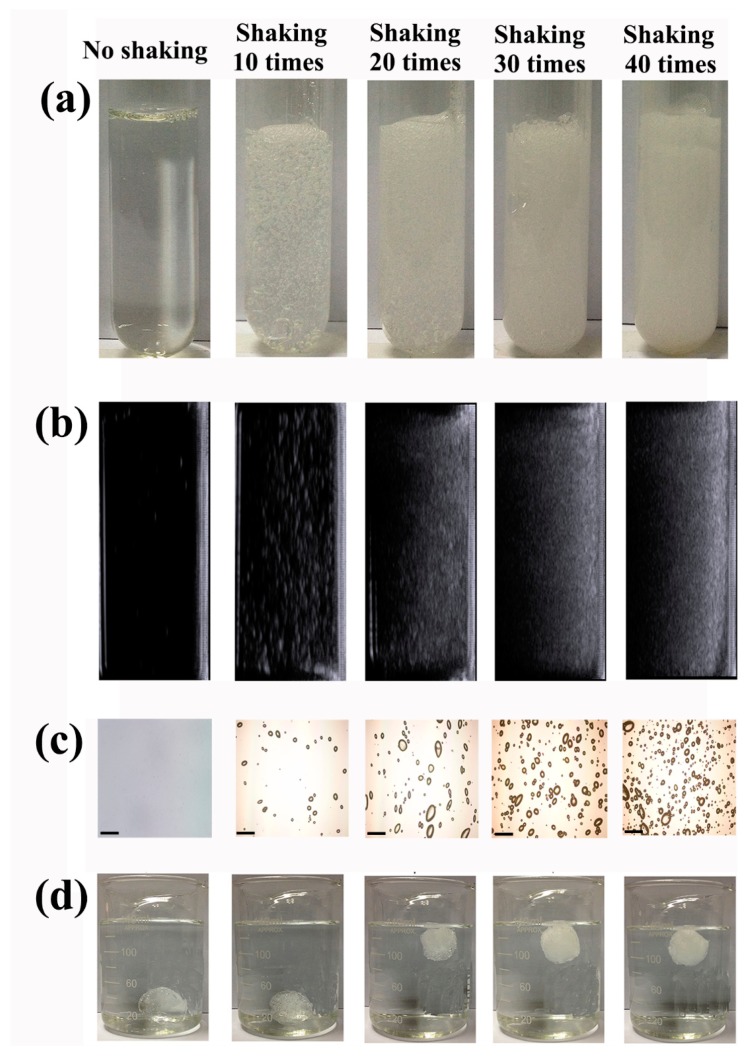
Effects of shaking time on bubbles in hydrogel: (**a**) bubbles in hydrogel solution recorded with photos; (**b**) bubbles in hydrogel solution detected by ultrasound; (**c**) bubbles in hydrogels under microscope; and (**d**) the floating state of hydrogels.

P407 was chosen as the matrix of floating hydrogel, and 45% (w/v) P407 was selected as optimal concentration according to our previous work [8]. As P407 was a kind of surfactant, the surfactant molecules could coat micro-bubbles by shaking, stirring, or homogenizing [9]. Enough micro-bubbles enlarge the volume of hydrogel and hence enhance the buoyancy, which eventually float the hydrogel. A large amount of micro-bubbles were produced in hydrogel solution after shaking for 40 times, while the hydrogel became opaque (Figure 2). To confirm that micro-bubbles were blocked in hydrogel at different temperature, hydrogel solutions after shaking were placed at 0 °C and 37 °C respectively. Results showed that the hydrogel could hold micro-bubbles in gel during the whole experiment at 37 °C (Figure 2a), while micro-bubbles dissipated gradually at 0 °C (Figure 2b). Because P407 is a thermo-sensitive material, it was gel state at 37 °C, but liquid state at 0 °C. Once the gel network formed, micro-bubbles was “locked” in hydrogel, and the hydrogel volume increased at the same time, so the buoyancy of hydrogel was increased and ensure the hydrogel stably float in liquid. We then investigate the effects of shaking time on generation of micro-bubbles. Hydrogel solution was shaken for 0, 10, 20, 30, 40 times respectively. Micro-bubbles produced in hydrogel solution were recorded with camera (Figure 3a). The density of micro-bubbles in gel increased with times of shaking. However, the hydrogel solution before shaking was transparent, with no micro-bubbles observed. To further observe the micro-bubbles produced in hydrogel solution, they were detected by ultrasound and images were captured (Figure 3b). As micro-bubbles in hydrogel was an ultrasound contrast agents, significant enhancement will be observed when a lot of micro-bubbles produced. The enhancement of hydrogel ultrasound images was also proportional to the shaking time. Similarly, the microscope images showed that the number of micro-bubbles in hydrogel increased with times of shaking (Figure 3c). According to these images, the mean number of micro-bubbles in hydrogel shaken for 10, 20, 30 and 40 times were 49 ± 5,107 ± 15, 165 ± 8 and 250 ± 16, respectively (Figure S2). To determine the volume change of hydrogel in respect to the density of micro-bubble, 10 mL of P407 solution was shaken by hand 0, 10, 20, 30, and 40 times. The volume changes of hydrogels after different shaking times were recorded. The mean volume change of hydrogel shaken for 10, 20, 30 and 40 times were 0.47 ± 0.15, 1.23 ± 0.25, 1.9 ± 0.20, and 2.23 ± 0.25, respectively (Figure S6), hydrogel volume increased indicated air gas incorporated into hydrogel and formed micro-bubbles. To determine the effect of environmental factors and shaking force on micro-bubble generation, the volume change of hydrogel in respect to the density of micro-bubble was measured. Results showed that environmental factors (temperature, humidity, and atmosphere pressure) had no significant effect on micro-bubble generation (Figure S7a–c). However, the hand shaking speed had great impact on micro-bubble generation of gel solution, shaking time greater than 1 s led to inadequate micro-bubbles generated for floating hydrogel (Figure S7d). To test the floating state of hydrogel in 37 °C water, these hydrogel solutions were put into 37 °C water. Results showed that the groups of hydrogels shaken over 20 times floated stably in water, other groups of hydrogel (not shaken or shaken 10 times) failed to float (Figure 3d). The erosion time and gelation temperature of hydrogels were also tested (Figure S1a,b). The gelation temperature of hydrogels shaken 10, 20, 30 and 40 times was 11.5 °C, 11.5 °C, 11.7 °C, 11.9 °C and 11.9 °C, respectively. The erosion time of non-floating hydrogel (not shaken or shaken 10 times) and floating hydrogel (shaken 20–40 times) were about 6.5 h and 6 h, respectively. The gelation temperature and erosion time of hydrogels showed no differences before and after shaking. These parameters indicated that the shaking did not affect the inherent property of hydrogel, but only floated it.

### 3.2. Characterization of Floating Hydrogel

**Figure 4:**
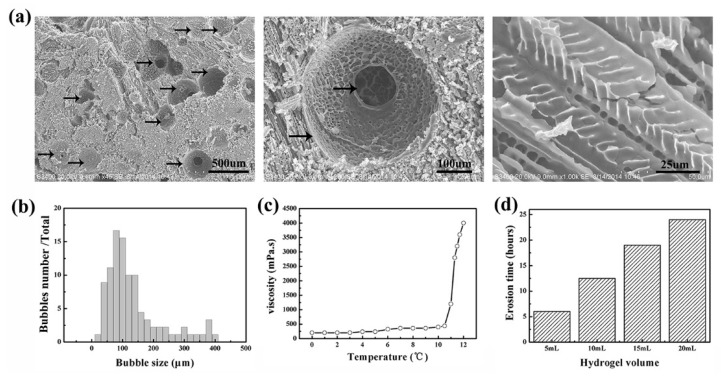
Characterizations of floating hydrogel: (**a**) the floating hydrogel morphology under SEM; (**b**) bubble size distribution of the floating hydrogel; (**c**) viscosity of the floating hydrogel; and (**d**) erosion time of the floating hydrogel with different volume.

The floating hydrogel morphology was investigated by SEM (Figure 4a). The floating hydrogel was porous, with clear polymer strands. Micro-bubbles produced in floating hydrogel could be seen in gel networks. The size of micro-bubbles in hydrogel ranged from 20 μm to 400 μm, most were 80~140 μm (Figure 4b). The viscosity of hydrogel increased drastically at 10.5 °C (its sol-gel transition temperature) and reached maximum at 12 °C (Figure 4c). To evaluate the effect of gel volume on hydrogels, we recorded the erosion time in aqueous solution. The erosion time of floating hydrogel increased with the hydrogel volume, ranging from 6 to 24 h as the hydrogel volume increased from 5 mL to 20 mL (Figure 4d). The storage stability of the hydrogel was also tested. The hydrogel could be stored up to nine months without significant change of the gelation. The drug entrapment efficiency was 100% due to the gelation of hydrogel.

### 3.3. Release Study In Vitro

**Figure 5:**
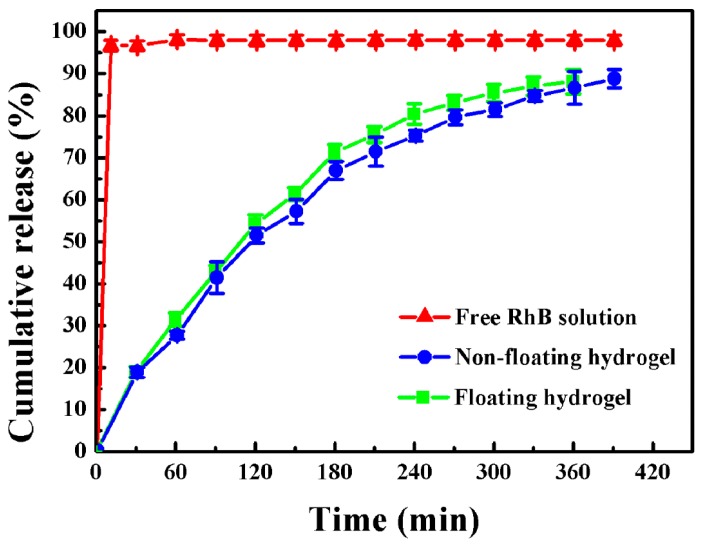
Cumulative release of free-RhB solution, RhB-loaded non-floating hydrogel and RhB-loaded floating hydrogel (Mean ± SD, *n* = 3).

The release profiles of free RhB solution, RhB-loaded non-floating hydrogel and RhB-loaded floating hydrogel in vitro were investigated (Figure 5). RhB served as fluorescence indicator. The curves depicted the cumulative release amount of RhB as a function of time. The control (free RhB) dispersed immediately to form homogenous solution in water, and the cumulative release reached 96.3% in 10 min after injection, while, after injecting RhB-loaded non-floating hydrogel or RhB-loaded floating hydrogel into water, floating hydrogel could float on the surface of water immediately. The time for RhB to be completely released from gel in the non-floating hydrogel group and floating hydrogel group were 6.5 and 6 h, respectively. We also found that the Higuchi model (Qt = K_H_$\sqrt{t}$) could predict the drug release from floating hydrogel perfectly in, which the release constant (K_H_) is 39.9 and the correlation coefficient (R) is 0.99 [12], which indicated that drug release from floating hydrogel was a controlled-release process and square root time dependent.

### 3.4. Release Study In Vivo

**Figure 6:**
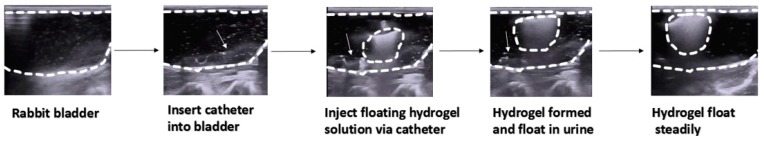
Process of intravesically injection of floating hydrogel into rabbit bladder (detected by ultrasound). The white dashed curves in the pictures represented the rabbit bladder wall. The white dashed circles represented gels in rabbit bladder. The catheters are indicated with white arrows.

Firstly, the process of intravesical injection of floating hydrogel into rabbit bladder was detected by ultrasound (Figure 6). Catheter was inserted into rabbit bladder via urethra, then the thermo-sensitive hydrogel solution was injected into bladder using catheter. After that, the hydrogel formed and floated in bladder immediately. More information about the floating process canbe seen in the Video S1 (Supplementary Materials). The ultrasound image of non-floating hydrogel in rabbit bladder was also captured (Figure S3). From the image, the non-floating hydrogel was unable to float in urine and attached to the bladder wall.

**Figure 7:**
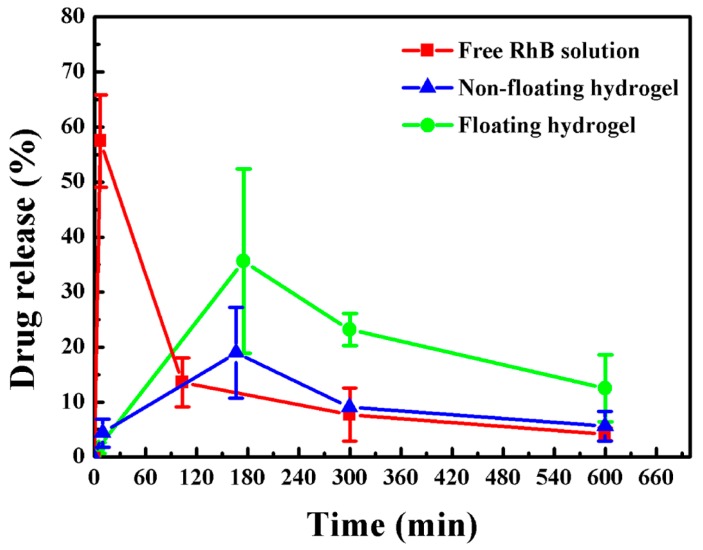
Drug release of floating hydrogel, non-floating hydrogel and free RhB solution in vivo (Mean ± SD, *n* = 3).

Then, we measured RhB release rate from non-floating and floating hydrogels, compared with free RhB in vivo (Figure 7). The first voiding of urine was triggered by filling the rabbit bladder 2~10 min after intravesical instillation of saline, free RhB solution, non-floating hydrogel or floating hydrogel (Figure S4). After intravesical instillation of RhB solution, RhB concentration in bladder reached the peak immediately, but dropped sharply after the first voiding of urine. As to the non-floating hydrogel group, due to the incapacity of floating in urine, it attached on the bladder wall, so it caused severe bladder irritation. Consequently, most gels were expelled from bladder during the first voiding of urine, and only little hydrogel remained. In contrast, the intravesical delivery of floating hydrogel showed sustained release of RhB from gel. The concentration of RhB released from floating hydrogel reached 35.6% before the second voiding. After voiding, floating hydrogel remained releasing drug, and RhB concentration reached 23.2% before the third voiding, and 12.5% before the last voiding. Compared to free RhB and non-floating hydrogel, floating hydrogel could significantly extend the residence time of RhB in bladder and hence increase the bioavailability.

**Figure 8:**
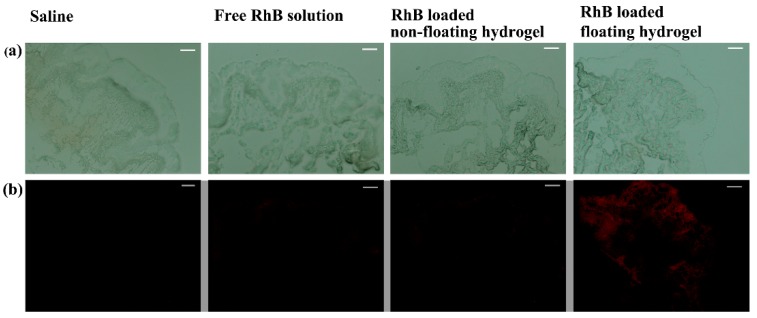
Phase and fluorescence picture of frozen sections of bladder tissue to evaluate residual of RhB in the bladder. (**a**) Bright-field of frozen sections of bladder tissue; (**b**) Fluorescence images of frozen sections of bladder tissue, the fluorescence intensity of floating hydrogel group was much higher than free RhB solution and non-floating hydrogel groups.

Finally, the residual amount of RhB in rabbit bladder of normal saline, free RhB solution, RhB-loaded non-floating hydrogel and RhB-loaded floating hydrogel groups were investigated (Figure 8). The results showed that the fluorescence intensity of floating hydrogel group was much higher than free RhB solution and non-floating hydrogel groups. No fluorescence of RhB could be observed in the normal saline group. The residual amount of RhB in the bladder tissue was proportional to the fluorescent intensity, so the residual amount of RhB in rabbit bladder of floating hydrogel group was much higher than other groups. Besides, there is no targeted delivery mechanism on the floating hydrogel drug delivery system selectively to the cancer cells rather than the healthy cells. Therefore, the floating hydrogel was a promising drug carrier for raising the efficiency of intravesical administration.

### 3.5. The Efficacy of Floating Hydrogel in Acute Bladder Injury Model

Interstitial cystitis (IC) patients have typical symptoms such as pain, frequency, urgency and nocturia, owing to damages to the glycosaminoglycan (GAG) layer of the bladder [13]. Acute bladder injury model induced by protamine sulfate was commonly used to study interstitial cystitis. Protamine sulfate destroyed the GAG layer of bladder mucosa, thus increasing the permeability of bladder mucosa. The protamine sulfate effect might be attenuated by exogenous sulfated polysaccharide, such as heparin.

**Figure 9:**
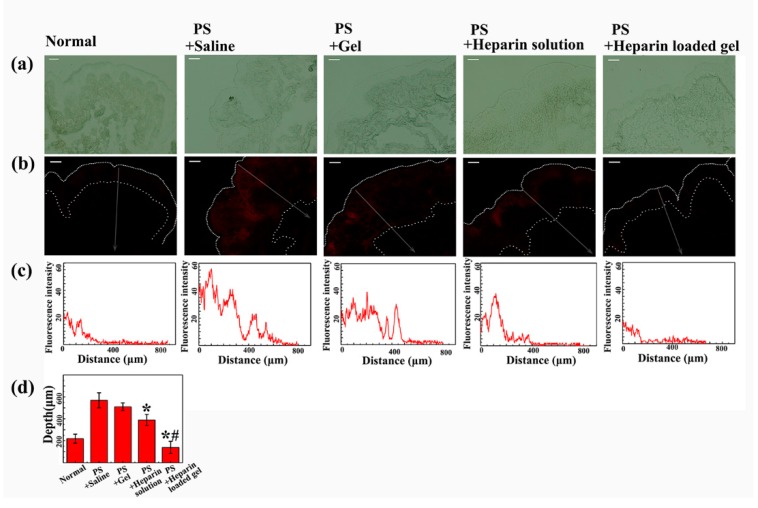
The efficacy of different treatments in acute bladder injury model: (**a**) phase picture of frozen sections of bladder tissue; (**b**) fluorescence picture of frozen sections of bladder tissue; (**c**) fluorescence intensity–distance profiles of different bladder frozen sections; and (**d**) penetration depth of different bladder frozen sections. (Mean ± SD, *n* = 3).

We investigated the efficacy of saline, floating hydrogel, heparin solution and heparin-loaded floating hydrogel groups in preventing the permeation of RhB in protamine sulfate pretreated bladders. The higher permeability of RhB indicated poorer efficacy of drugs. In our experiment, the permeability of bladder mucosa was evaluated by the fluorescence depth of RhB in bladder wall (Figure 9). The fluorescence depth was indicated by the width of fluorescence zone in the bladder wall, and the fluorescence distribution with width was also plotted (Figure 9b,c). Figure 9d showed that the fluorescence depth of RhB in the PS + saline group and PS + Gel group were much larger than the other two groups (*p* < 0.05), proving the capacity of heparin to reverse the wall leakage that PS caused. Compared to PS + heparin solution group, PS + heparin-loaded gel group showed much poorer permeation of RhB (*p* < 0.05), with width similar to control group, indicating better efficiency of heparin. Because the time of first voiding of all groups were 2~10 min, and the heparin solution was eliminated during that time. However, floating hydrogel could stay in bladder and continued to release heparin, largely improving the efficacy of heparin. Therefore, the floating hydrogel system could successfully extend the residence time of drug and hence enhance drug efficacy.

## 4. Discussion

The safety problem of medical material is a tough obstacle lying in its application from bench to bedside. In this study, we chose Poloxamer 407 as the only ingredient of our delivery system. The safety of P407 hydrogel in vivo has been widely studied by previous reports [14]. In terms of biocompatibility studies in vivo, there was no damage observed in muscle cells after injection of 20% P407 [15]. Moreover, after intravesical instillation, the concentration of P407 in urine was much lower than 20%. Furthermore, preparation of this system was just shaking the P407 aqueous solution for 20 s, which is extremely simple. Therefore, there is no safety problem in the clinical application of our system. The efficacy of our system is inspiring according to in vivo drug release and efficacy experiments, much better than non-floating hydrogels and drug solutions.

P407 is polyoxyethylene-polyoxypropylene-polyoxyethylene (PEOn-PPOn-PEOn) tri-block copolymer, non-ionic surfactants, possessing biocompatible, thermo-sensitive, low toxicity, foamability and weak immunogenic properties [14,16]. In this study, its foamability enabled P407 to act as micro-bubbles source of the floating hydrogel system. By shaking the P407 solution, air was incorporated in the solution, a bubble rising in a surfactant solution accumulates surfactant molecules on its surface (the surfactant adsorption at the air/water interface) [17]. The number or growth rate of micro-bubbles increased with the concentration of surfactant (P407) in solution, as well as air amount incorporated in the solution (i.e., more shaking times) [18]. The stability of micro-bubbles in solution was positively correlated with the concentration or viscosity of surfactant [19]. Thus, micro-bubbles would suspend in solution for a period because of the viscosity of the P407 solution. When P407 solution converted to gel state (at 37 °C), so the air micro-bubbles in gel could not move any more thus the micro-bubbles were “locked” in gel. When the gel changed to the solution state (at 0 °C), the air micro-bubbles in solution would gradually dissipate away because of the stochastic nature of bubble coalescence in viscous liquids. We also have tested the gelation properties of the solution at 25 °C, the gelation temperature of the hydrogel solution was about 11.5 °C, so the hydrogel converted to the gel state from the solution state at 25 °C. Since in clinical operation, the injection will be operated under room temperature, so we stored the gel solution at 0 °C condition before injected to the bladder, the viscosity of 45% P407 was low (about 200 mPa·s) at this temperature. When take the gel solution under room temperature from 0 °C condition, the gelation time of the gel solution was about 5 min after shaking quickly. Therefore, it has enough time to inject the gel solution into bladder. As the shear force have impact on the stability of the micro-bubbles during injection, we injected the hydrogel solution (3 mL) into the bladder quickly (about 10 s). The short injection time may reduce the impact of shear force. The floating state of hydrogel was confirmed by ultrasound after injection. We also used homogenizer to produce micro-bubbles. Hydrogel solution stirred for 10 s at the speed of 5000 rpm could float in water stably (Figure S5). Considering the convenience in clinical application, we used shaking by hand to replace machine.

The time to float of hydrogel should be as short as possible since the short time can decrease the risk of urinary obstruction or bladder irritation. In this floating hydrogel system, the hydrogel could float immediately due to the whole hydrogel was full of micro-bubbles at the beginning by shaking the P407 solution. However, the time to float of hydrogels we previously developed were several minutes, because chemical reaction took time [7,8].

P407 formed micelles to entrap drugs, so drugs was released throughout the diffusion process of gel matrix. Drugs released with the process of P407 dissolution and ended once P407 dissolved completely [20]. The erosion time of hydrogel should be longer to extend the release time of drug. Increasing concentration and hydrogel volume of P407 will lengthen its erosion time and hence extend the release time of drug. Moreover, the viscosity of gels also increased with the concentration of the P407 [20]. Therefore, 45% P407 was chosen to obtain optimal erosion time and viscosity. The results showed that the erosion time of this floating hydrogel system (about 6 h/5 mL) was much longer than our previous floating hydrogel containing NH_4_HCO_3_ (about 3 h/5 mL) [8]. These are due to the chemical reaction inside hydrogel accelerated the gel erosion.

In this study, the drug release time in vivo was not consistent with that in vitro. The erosion time of the 3 mL floating hydrogel was about 3.5~4 h in vitro. However, in vivo release study showed that drug was released for about 10 h. The time discrepancy of drug release might result from the volume difference between bladder (5~40 mL) and beaker (200 mL). Another reason was the urine residues in the bladder [21]. In addition, the autofluorescence of rabbit urine might increase the measurement of RhB release amount. Moreover, the contraction of bladder during injection and gelation process will reduce the amount of micro-bubble generated and thus result in the reduction in erosion time in vivo.

Bladder irritation is often induced by urinary infection or foreign bodies in the bladder (such as bladder stone or blood clots) [22]. In our experiments, most non-floating hydrogels were expelled from rabbit bladder after intravesical instillation. It indicated that non-floating hydrogel caused severe bladder irritation as it attached to the bladder wall, and caused the rabbit bladder detrusor forcefully contracted to expel it out. Compared to the non-floating hydrogel, the floating hydrogel was floating in urine and was not directly in contact with the bladder wall, so the floating hydrogel could avoid cause the bladder irritation.

Previous reports have shown that patients of interstitial cystitis endure damage of the glycosaminoglycan (GAG) layer of urothelium [23]. The surface GAG layer has been proposed as a protective barrier that coats the transitional cell surface. Parsons has demonstrated that the increasing bladder mucosal permeability to solu-urea in patients with interstitial cystitis could be imitated by intravesical administration of protamine sulfate [24]. The protamine effect appeared to be reversed by treatments with exogenous sulfated polysaccharide (i.e., intravesical administration of heparin, hyaluronic acid, or pentosan polysulfate) [25]. Heparin was initially reported to relieve the symptoms of IC in the 1960s, and subsequent studies also proved its efficacy [26]. Its exact mechanism of action was not understood at that time. One hypothesis was that heparin provided exogenous sulfated polysaccharide which coated the epithelium to maintain the bladder mucosa integrity. In this study, rabbit acute bladder injury model was constructed by intravesical administration of protamine sulfate, and the efficacy of heparin loaded floating hydrogel was evaluated by fluorescence depth in the bladder wall. Our results showed that the heparin-loaded floating hydrogel was more effective than free heparin solution in alleviating the damage effects of protamine sulfate. Because the floating hydrogel released RhB persistently and extended the residence time of heparin in bladder, while the free heparin solution was eliminated of bladder along with the first voiding of urine. Our results also showed that the P407 hydrogel did not affect the bladder mucosal permeability to RhB. As the heparin served as the exogenous sulfated polysaccharide, the prolonged drug resident time in bladder increased the efficiency of heparin in the protection of bladder mucosal permeability. The floating hydrogel system was a promising drug delivery system in the treatment of IC patients whose bladder GAGs layer was damaged.

## 5. Conclusions

This paper presents a new self-generating micro-bubbles floating hydrogel drug delivery system for intravesical instillation. The Matrix of floating hydrogel is P407 without any additives, addressing safety concerns for clinical use. The floating property of floating hydrogel avoids urinary obstruction and bladder irritation that conventional hydrogels cause, and improves drug efficacy by prolonging the residence time. In other words, this new drug delivery system is safe, simply prepared and efficient for future clinical use.

## Supplementary Materials

The following are available online at www.mdpi.com/1996-1944/9/12/1005/s1. Figure S1: Effects of shaking time on erosion time (a) and gelation temperature (b) in hydrogel, Figure S2: Effects of shaking time on micro-bubbles number in hydrogel, Figure S3: Non-floating hydrogel in rabbit bladder, Figure S4: The time of first voiding after intravesical instillation, Figure S5: Hydrogel solution was homogenized, then hydrogel floats stably in water, Figure S6: Volume change of hydrogel in respect to the density of micro-bubble, Figure S7: Effect of environmental factors (temperature (a), humidity (b), and atmosphere pressure (c)) and the shaking force (d) on micro-bubble generation, Video S1: Floating hydrogel in vitro and vivo.
