# Supplementary material for: Floating Hydrogel with Self-Generating Micro-Bubbles for Intravesical Instillation
Source: Materials (Basel). 2016 Dec 12;9(12):1005. doi: 10.3390/ma9121005 (PMC5456973; doi:10.3390/ma9121005)
Supplement: Supplementary file 1 [file materials-09-01005-s001.pdf]

Supplemental Materials: Floating Hydrogel with Self-Generating Micro-Bubbles for Intravesical Instillation

Tingsheng Lin, Xiaozhi Zhao, Yifan Zhang, Huibo Lian, Junlong Zhuang, Qing Zhang, Wei Chen, Wei Wang, Guangxiang Liu, Suhan Guo, Jinhui Wu, Yiqiao Hu and Hongqian Guo

Measurements of erosion time and gelation temperature

The erosion time was the time it takes for gel to dissolve completely in water. The hydrogels (the volume of hydrogels were all 5 mL) were shaken manually for 0, 10, 20, 30, 40 times, respectively. Then hydrogels were injected into 37 °C water, and the erosion time of floating hydrogels was recorded.

The gelation temperature was the lowest temperature at which P407 solution converted to gel. The tube inversion was used to determine the gelation temperature [1].

Bubbles number of floating hydrogels

To evaluate the effect of shaking time on micro-bubbles amount in hydrogel, P407 solution was shaken by hand for 0, 10, 20, 30, 40 times respectively and placed at ambient temperature to form gel. Hydrogels were put on a glass slide and observed by a Zeiss M2Bio microscope. The amplification factor of microscope was 40×. Three fields were randomly selected to calculate the bubbles number.

Volume changes of hydrogels after different shaking times

To determine the volume change of hydrogel in respect to the density of micro bubble, 10 mL of P407 solution was shaken by hand for 0, 10, 20, 30, 40 times respectively. The volume changes of hydrogels after different shaking times were recorded.

Effect of environmental factors and shaking force on micro bubble generation.

To determine the effect of environmental factors and shaking force on micro bubble generation, the volume change of hydrogel in respect to the density of micro bubble was measured. 10 mL of P407 solution was shaken by hand for 20 times at different environmental factors and the volume changes of hydrogels after shaking were recorded. To observe environmental effect, gel solutions were shaking at the local different temperature (3.5 °C, 11.5 °C, 25.3 °C and 31.9 °C), local different humidity (22.6%, 46.8%, 69.4% and 88.2%), local different atmosphere pressure (1005/100 Pa, 1009/100 Pa, 1013/100 Pa and 1015/100 Pa). To evaluate shaking force on micro bubble generation, shaking speed in respect to the shaking force was measured. Shaking speed defined as the time of hand move up and down in one cycle (hand move up and down was one cycle, the distance of hand up or down was 50 cm).

Detection of non-floating hydrogel in rabbit bladder using Ultrasound

Rabbits were anesthetized with pentobarbital (30 mg/kg, intravenous injection). Hydrogel solution without shaking was intravesically instilled into bladder using a catheter. The hydrogel in bladder was detected by B ultrasound.

The time of first voiding after intravesial instillation

The first voiding of urine was triggered by filling the rabbit bladders with saline after intravesical instillation, and the time of first voiding of urine were recorded.

Micro-bubbles produced in hydrogel solution using homogenizer

Micro-bubbles also could be produced in hydrogel solution using homogenizer (IKA T25, Germany). 10 mL hydrogel solution was placed in a glass tube, then hydrogel solution stirred for 10 s at the speed of 5000 rpm.

Illustrations for Figure 9b

The outside white dash line represented bladder mucosal surface, the inside white dash line represented the fluorescence when disappeared in bladder tissue, the white arrows was the trace that measured the fluorescence intensity.

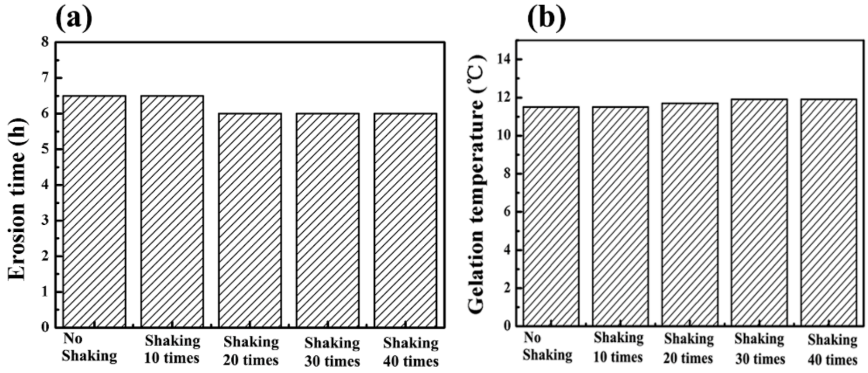

Figure S1. Effects of shaking time on erosion time (**a**) and gelation temperature (**b**) in hydrogel.

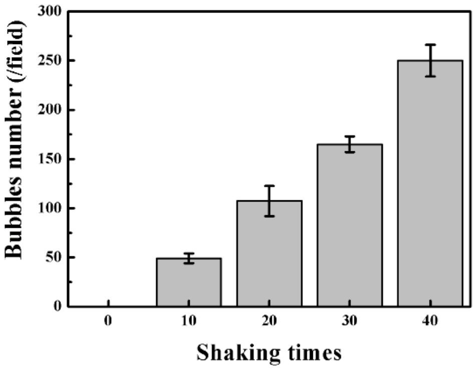

Figure S2. Effects of shaking time on micro-bubbles number in hydrogel.

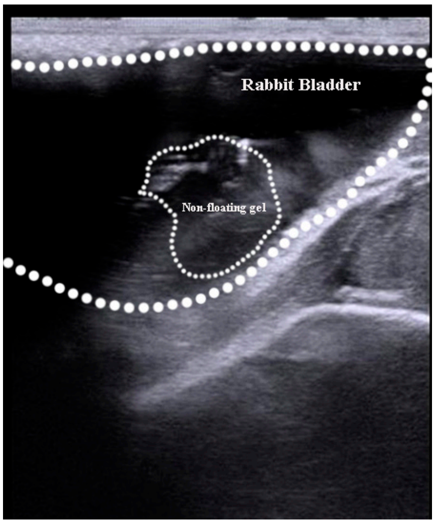

Figure S3. Non-floating hydrogel in rabbit bladder.

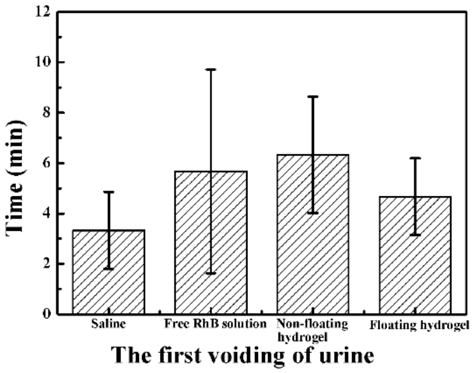

Figure S4. The time of first voiding after intravesial instillation.

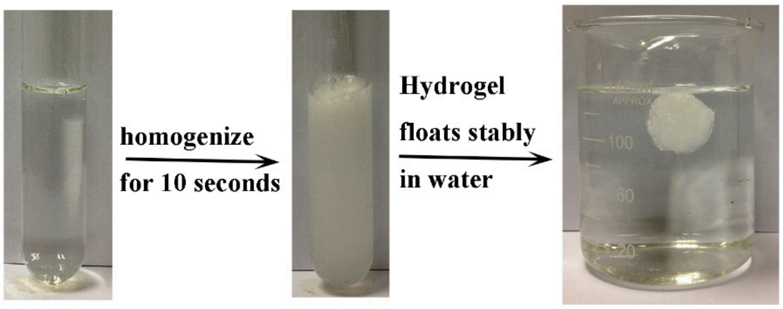

Figure S5. Hydrogel solution was homogenized, then hydrogel floats stably in water.

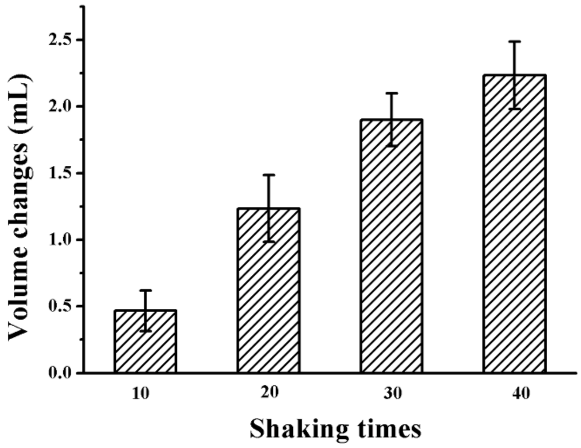

Figure S6. Volume change of hydrogel in respect to the density of micro bubble.

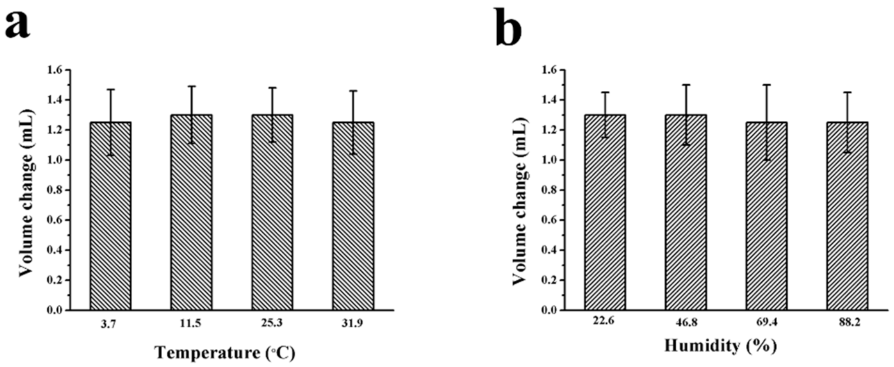

Figure S7. *Cont.*

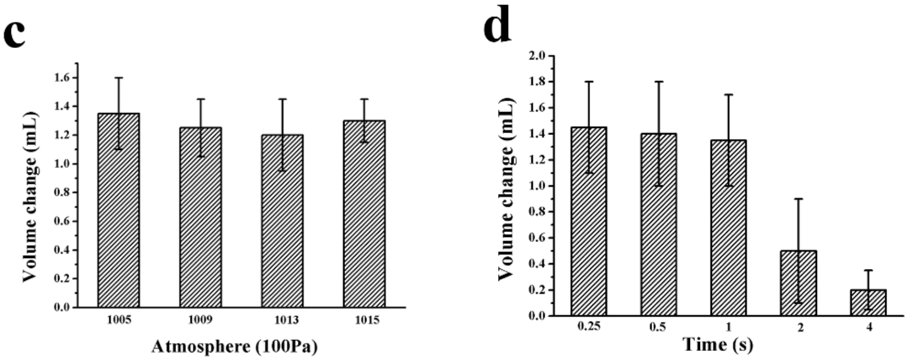

Figure S7. Effect of environmental factors (temperature (**a**), humidity (**b**), and atmosphere pressure (**c**)) and the shaking force (**d**) on micro bubble generation.

Reference

1. Sharma, P.K.; Bhatia, S.R. Effect of anti-inflammatories on Pluronic F127: Micellar assembly, gelation and partitioning. *Int. J. Pharm.* **2004**, *278*, 361–377.
